# Longitudinal assessment of renal size and function in extremely low birth weight children at 7 and 11 years of age

**DOI:** 10.1007/s00467-016-3413-6

**Published:** 2016-05-27

**Authors:** Katarzyna Starzec, Małgorzata Klimek, Andrzej Grudzień, Mateusz Jagła, Przemko Kwinta

**Affiliations:** Department of Pediatrics, Jagiellonian University Medical College, Wielicka Street 265, Cracow, 30-663 Poland

**Keywords:** Preterm infants, ELBW, Cystain C, eGFR, Renal ultrasound, Renal volume, Renal function, Renal complications

## Abstract

**Background:**

There are a lack of studies describing a longitudinal association between preterm delivery and renal complications later in life. We assessed renal size and function in preterm infants born with extremely low birth weight (ELBW) during 4 years of follow-up, comparing these parameters to age-matched children born full term (term controls).

**Methods:**

The results of selected renal laboratory tests [levels of cystatin C, creatinine, blood urea nitrogen (BUN)] and of renal ultrasound evaluations were compared between the ELBW group and the term control group at age 7 and 11 years.

**Results:**

The study population consisted of 64 children born with ELBW (ELBW children) who had been recruited at birth and 36 children born at term (term children) who took part in both follow-up assessments. Renal ultrasound examination revealed a significantly smaller renal volume in the 7- and 11-year-old ELBW children compared to the term controls [right kidney volume: 50.8 vs. 61.2 ml/m^2^, respectively, at 7 years (*p* <0.01) and 51.4 vs. 58.2 ml/m^2^, respectively, at 11 years (*p* <0.01); left kidney volume: 51.4 vs. 60.3 ml/m^2^, respectively, at 7 years (*p* <0.01) and 55.2 vs. 60.7 ml/m^2^, respectively, at 11 years (*p* = 0.02)]. Renal function in ELBW children was also affected. Serum cystatin C levels were significantly higher in ELBW children than in the controls at 7 years of age, and this difference remained statistically significant at 11 years of age [0.63 vs. 0.59 mg/l, respectively, at 7 years (*p* = 0.02) and 0.72 vs. 0.61 mg/l, respectively, at 11 years (*p* = 0.01)]. Six ELBW children also had elevated cystatin C levels (0.97–1.11 mg/l) at 11 years of age. Cystatin C levels were within normal range in the ELBW children at age 7 years and in term children in both follow-up studies. BUN levels were higher in ELBW children at the age of 11 years (4.49 vs. 4.15 mmol/l; *p* = 0.028).

**Conclusion:**

Continued follow-up of these patients will reveal whether the observed worsening in renal function will persist into adulthood.

## Introduction

Over the past three decades the survival of extremely preterm infants, including those born with an extremely low birth weight (ELBW) (defined as an infant weighing <1000 g at birth), has improved significantly, largely due to improvements in the quality of prenatal, perinatal and neonatal care [1–3]. However, many of these infants develop long-term complications as a consequence of acute complications which have their origin during the perinatal period [1, 4–6]. Many of the studies reported in the literature on this subject have focused on neurological and cardiovascular complications, including developmental delay, psychomotor dysfunction, psychiatric disorders, autism and learning disabilities [7–16]. More recently, however, an increasing number of studies are examining the association between preterm delivery, nephron number, renal size, microalbuminuria and risk of hypertension in adult life [17, 18]. From the physiological perspective, nephrogenesis is an ongoing developmental process that continues until 34–36 weeks’ gestation; consequently, very prematurely born neonates (gestational age <32 weeks) are likely to have a reduced number of nephrons at birth. This deficit in nephron number has been found in several studies. Rodriguez et al. demonstrated in an autopsy study that premature ELBW infants (gestational age <28 weeks, birth weight ≤1000 g) had markedly fewer radial glomerular counts than term infants [19]. These authors also found that nephron number was highly correlated with gestational age. Other researchers have reported that although glomerulogenesis continues until day 40 of postnatal age it never reaches the level seen in term infants [20, 21]. Many ELBW infants are also exposed to other risk factors associated with impaired postnatal renal development, such as hypoxia, hyperoxia, use of nephrotoxic medications, acute kidney injury, nephrocalcinosis and poor intra- and extrauterine nutrition [22–27]. Decreased renal size has been described in adults born very preterm compared to those born at full term. Authors focusing on the renal complications of preterm birth emphasize the need for a prolonged follow-up period for children born with ELBW (referred to hereafter as ELBW children) [28–30].

The aim of our study was to assess renal size and function in school-age ELBW children compared to their peers and its evolution within a 4-year observation period. We compared the results of selected renal function laboratory tests and of ultrasound examinations between 11-year-old ELBW children and age-matched children born at full term (referred to hereafter as term children). We then assessed the variability of the measured parameters over time in the ELBW group to establish a correlation between extremely preterm delivery and kidney growth and function.

## Methods

A cross-sectional observational study was conducted in the outpatient paediatric department of the Polish-American Children’s Hospital in Krakow, Poland, between 30 December 2013 and 30 April 2015. The study cohort consisted of a study group composed of ELBW children recruited at birth (ELBW group) and a control group composed of composed of children enrolled in the study at 6–7 years of age (term group). From 1 September 2002 to 31 August 31 2004, 169 newborns with a birth weight of <1000 g were born alive in the southeast district of Poland (Malopolska Region). All children were hospitalized in one of three tertiary neonatal intensive care units (NICUs) in southeast Poland. Ninety-one infants were discharged home from these NICUs and followed longitudinally. All children who took part in the follow-up assessment at 6–7 years of age were invited to participate in the present follow-up study as well. Neonatal data used for the study were recorded daily during the hospitalization of each infant in the NICU in a prospective manner and stored on computer databases. The age-matched term control group was formed in 2009–2010 and consisted of 38 children born with a birth weight of >2500 g who were recruited from one general practitioner’s office. All of these children were invited for a re-assessment later in the study. The methodology and results of the follow-up study performed when the children were 6–7 years of age have already been published [31].

### Follow-up at 10–11 years of age

After providing informed consent, all participants underwent a physical examination consisting of anthropometric measurements, blood sampling for the assessment of cystatin C, creatinine and blood urea nitrogen (BUN) levels and an ultrasound examination of the abdomen. The parents were also ask to complete a questionnaire on their child’s demographic data and health status. Both the physical examination and completion of the questionnaire occurred during a single visit to the paediatric outpatient department of the Polish-American Children’s Hospital of Krakow.

### Anthropometric measurements

Body height was measured to an accuracy of 1 mm using a stadiometer with the patients standing in an upright position with their heads in the ocular-ear position. Body weight was measured as previously described [32]. For each patient the standard deviation of height and weight was calculated to two decimal points, and body surface area (BSA) was calculated using the formula provided by Du Bois and Du Bois: BSA = weight (kg) ^0.425^ × height (cm) ^0.725^ × 0.007184.

### Serum cystatin C levels

Cystatin C was measured by a particle-enhanced immunonephelometric assay (N Latex Cystatin C assay; Dade Behring, Deerfield, IL) using a nephelometer (BN-II; Dade Behring). The normal value for children between 4 and 12 years of age was defined as 0.53–0.95 mg/l. The same method was used to evaluate cystatin C levels in children 7 and 11 years of age.

### Creatinine and BUN

Creatinine and BUN were measured by using VITROS® Chemistry Products (Ortho Clinical Diagnostics, Raritan, NJ). The two-point rate test for creatinine and the colorimetric test for BUN were performed using the VITROS® 5,1 FS Chemistry System, 950, 750, 550, 250. The normal range of creatinine and BUN in children aged 10 and 11 years was defined as 6.0–74.0 umol/l and 2.5–6.0 mmol/l, respectively.

### Estimated glomerular filtration rate

Estimated glomerular filtration rate (eGFR) was calculated by using the formula provided by Hoek et al.: eGFR = −4.32 + (80.35/Cys) [33].

### Renal ultrasound

Each child had two renal ultrasound examinations (Philips EnVisor C HD system with color Doppler and C2-5 MHz probe; Koninklijke Philips N.V., Eindhoven, the Netherlands) at ages 7 and 11 years, respectively; all examinations were performed by the same two examiners using the same protocol for both follow–up evaluations. For each child, kidney length and width were calculated as the average of three measurements. Renal measurements were performed with the subject lying in the supine position and scanned in the para-coronal view with the transducer positioned to obtain the longest kidney dimension. Kidney volume was calculated using the formula: (kidney length × kidney width × kidney thickness) × Pi/6. Relative kidney length was obtained by calculating the ratio of the mean actual measurement to the mean renal reference measurement. The reference values of kidney length were calculated using the formula of Dinkel et al. [34]. Renal length was adjusted to the patient’s height. The formula used to calculate predicted renal length were:

$$ \begin{array}{l}\mathrm{Left}\ \mathrm{kidney}\left(\mathrm{mm}\right):\ 0.51346 \times \mathrm{height}\left(\mathrm{cm}\right) + 17.659\hfill \\ {}\mathrm{Right}\ \mathrm{kidney}\left(\mathrm{mm}\right):\ 0.49915 \times \mathrm{height}\ \left(\mathrm{cm}\right) + 18.381\hfill \end{array} $$

The formula used to calculate relative kidney length was:

$$ \mathrm{Relative}\ \mathrm{kidney}\ \mathrm{length} = \mathrm{actual}\ \mathrm{kidney}\ \mathrm{length}\ \mathrm{measured}\ \mathrm{b}\mathrm{y}\ \mathrm{ultrasound}\ /\ \mathrm{predicted}\ \mathrm{kidney}\ \mathrm{length} $$

Relative kidney volume was established by dividing the ultrasonographic measurements by the BSA according to Scholbach and Weitzel [35]. The same method was used in both groups (ELBW and term) at 7 and 11 years of age.

### Outcome variables

Primary outcomes at 10–11 years of age were: (1) presence of small kidneys, defined as a relative kidney length of <75 % or relative kidney volume of <45 ml/m^2^; (2) presence of asymmetric kidneys, defined as a difference between relative kidney volumes of >20 %; (3) abnormal serum cystatin C levels (>0.95 mg/l). Secondary outcome variables were absolute and relative kidney length and volume, and serum cystatin C levels. The results obtained for the children at aged 10–11 years were then compared to those obtained during the first follow-up visit (at age 6–7 years).

### Statistical methods

To compare the ELBW and term control groups, we used the following tests as deemed appropriate: Mann–Whitney *U* test, Fisher’s exact test, Pearson’s chi-squared test, Welch’s *t* test, Student’s *t* test, Wilcoxon signed rank test and Paired *t* test. Statistical significance was defined at the *p* = 0.05 level for the two-sided test. Data were analysed using SPSS version 22 (2013) software (IBM Corp., Armonk, NY).

## Results

### Population

The study group was composed of 64 children born prematurely (among the 78 children who participated in the first follow-up study at 7 years of age), and the control group was composed of 36 children born at full term (among the 38 children who participated in the first follow-up study at 7 years of age). The characteristics of the ELBW group are presented in Table 1. Comparisons of the selected demographic and clinical variables between the ELBW and control groups are presented in Table 2.

**Table 1:** Characteristics of the children born with extremely low birth weight

Characteristics of the ELBW group	Values
Birth weight (g)	875 (750–960)
Gestational age (weeks)	27 (25–28)
Five-minute Apgar score	6 (5–7)
Small for gestational age	19 (30 %)
Treatment with prenatal steroids	31 (48 %)
Mechanical ventilation	58 (91 %)
Administration of surfactant	49 (77 %)
PDA treatment	19 (30 %)
Oxygen on 28th day of life	44 (69 %)
Oxygen at 36 weeks postmenstrual age	26 (41 %)
IVH grade III or IV	6 (9 %)
Weight gain during NICU stay	18 (15–23)
Length of hospitalization (days)	81 (56–106)

Data are presented as the number of patients with the percentage given in parenthesis or as the median with the 25th-75th percentile [interquartile range (IQR)] given in parenthesis, as appropriate

ELBW, Extremely low birth weight; PDA, patent ductus arteriosus; IVH, intraventricular haemorrhage; NICU, neonatal intensive care uni)

**Table 2:** Comparison of selected demographic and clinical variables between the infants born with extremely low birth weight (ELBW children) and infants born at term (control children)

Demographic and clinical variables	Control (full term) group (*n* = 36)	Study (ELBW) group (*n* = 64)	*p* value^a^
Demographic variables			
Female	17 (47 %)	43 (67 %)	0.06^b^
Birth weight (g)	3570 (3395–3880)	875 (750–960)	<0.01^c^
Gestational age (weeks))	40 (39–40)	27 (25–28)	<0.01^c^
Vaginal delivery	31 (86 %)	13 (20 %)	<0.01^b^
Multiple pregnancy	0	9 (14 %)	0.024^b^
Small for gestational age	2 (6 %)	19 (30 %)	0.004^b^
First follow-up (at age 7 years)			
Age at evaluation (years); median (25th-75th percentile)	6.9 (6.4–7.4)	6.5 (6.4–6.8)	0.04^c^
Height (cm)	124 ± 7.4	116 ± 6.3	<0.01^d^
Height (*z* score)	0.21 ± 1.0	−1.08 ± 1.3	<0.01^d^
Weight (kg)	25.2 ± 5.3	19.5 ± 3.8	<0.01^d^
Weight (*z* score)	0.26 ± 1.2	−0.92 ± 1.3	<0.01^d^
Second follow-up (at age 11 years)			
Age at evaluation (years)	10.7 (10.2–11.1)	11 (10.8–11.3)	0.01^c^
Height (cm)	146 ± 8	141 ± 8	<0.01^d^
Height (*z* score)	0.20 ± 1.1	−0.88 ± 1.2	<0.01^d^
Weight (kg)	40.4 ± 9.9	33.7 ± 8.2	<0.01^d^
Weight (*z* score	0.24 ± 1.2	−0.92 ± 1.3	<0.01^d^

Data are presented as the number of patients with the percentage given in parenthesis, the median with the IQR given in parenthesis and the mean ± standard deviation (SD), as appropriate

^a^Statistical significance was defined at the *p* = 0.05 level for the two-sided test

^b^ *p* value using the Fisher exact test

^c^ *p* value using the Mann–Whitney *U* test

^d^ *p* value using Student’s *t* test

Children born prematurely were significantly shorter than their peers born at term, with the mean height difference at 7 years of age reaching 1 standard deviation (*z* score −1.08 vs. 0.21;* p* <0.01) and remaining at this level 4 years later (*z* score −0.88 vs. 0.20;* p* <0.01). The weight of the preterm children was also significantly lower than that of their peers born at term (7 years:* z*-score −0.92 vs. 0.26,* p* <0.01; 11 years:* z*-score −0.92 vs. 0.24,* p* <0.01). Based on these observations, all kidney measurements were corrected to patient’s length (kidney length) or BSA (kidney volume).

### Cystatin C, creatinine and BUN levels

Serum cystatin C levels were significantly higher in the ELBW group than in the control group at 7 years of age (0.63 vs. 0.59 mg/l, respectively; *p* = 0.02), and this difference remained statistically significant at 11 years of age (0.72 vs. 0.61 mg/l, respectively; *p* = 0.01). Cystatin C levels were within the normal range in all ELBW children at 7 years of age; however, six ELBW children had elevated cystatin C levels (0.97–1.11 mg/l) at 11 years of age (Table 3). Cystatin C levels were higher in ELBW children at 11 years of age than at 7 years of age, and this difference was statistically significant (Table 4). Cystatin C levels were normal in the control children in both follow-up studies.

**Table 3:** Primary outcome variables for extremely low birth weight (ELBW) children and control children

Primary outcome variables	Control (full term) group (*n* = 36)	Study (ELBW) group (*n* = 64)	*p* value according to the Fisher exact test
First follow-up (at age 7 years)			
Relative kidney length <75 % of normal value	0	1	1.0
Relative kidney volume < 45 ml/m^2^	1	21	<0.01
Asymmetric kidneys (difference > 20 %)	1	12	0.02
High serum cystatin C level (>0.95 mg/l)	0	0	1.0
Second follow-up (at the age of 11 years)			
Relative kidney length <75 % of normal value	0	3	0.55
Relative kidney volume < 45 ml/m^2^	1	19	<0.01
Asymmetric kidneys (difference > 20 %)	3	8	0.7
High serum cystatin C level (>0.95 mg/l)	0	6	0.08

Data are presented as the number of children

**Table 4:** Results of paired *t*-test for selected biochemical and ultrasonographic measurements

Selected biochemical and ultrasonographic measurements	Control (full term) group (*n* = 36)	*p* according to paired *t* test	Study (ELBW) group (*n* = 64)	*p* according to paired *t* test
Relative right kidney volume difference between 1st and 2nd follow-up (ml/m^2^)	−2.6 (−7.4 to −2.01)	0.25	0.33 (−2.6 to 3.3)	0.82
Relative left kidney volume difference between 1st and 2nd follow-up (ml/m^2^)	0.82 (−4.02 to 5.7)	0.78	3.7 (−0.4 to 7)	0.11
Cystatin C difference between 1st and 2nd follow-up (mg/l)	0.01 (−0.03 to 0.06)	0.51	0.10 (0.05–0.14)	<0.001

Data are presented as the mean difference with the 95 % confidence interval (CI) for difference given in parenthesis

The BUN and creatinine levels were not measured in children at 7 years of age. However, they were measured in all children at 11 years of age, revealing significantly higher BUN levels in the ELBW children than in the term controls (4.49 vs. 4.15 mmol/l, respectively; *p* = 0.028) (Table 5). The difference in serum creatinine levels between the ELBW and term control children at 11 years of age was not statistically significant (Table 5).

**Table 5:** Comparison of the results of renal function tests between extremely low birth weight (ELBW) children and control children

Renal function tests	Control (full term) group (*n* = 36)	Study (ELBW) group (*n* = 64)	*p* value according to Student’s *t* test
First follow-up (at age 7 years)			
Serum cystatin C level (mg/l)	0.59 ± 0.08	0.63 ± 0.07	0.02
Second follow-up (at age 11 years)			
Serum Cystatin C level (mg/l)	0.61 ± 0.08	0.72 ± 0.15	0.01
BUN (mmol/l)	4.15 ± 0.94	4.49 ± 1.03	0.028
Creatinine (umol/l)	46.3 ± 7.6	43.2 ± 7.7	0.08

Data are presented as the mean ± SD

BUN, Blood urea nitrogen

### Estimated glomerular filtration rate

The eGFR values of children at 7 and 11 years of age were significantly lower in the study (ELBW) group than in the (full term) control group (*p* = 0.0116 at 7 years and *p* = 0.0001 at 11) years. Comparison of the eGFR in ELBW children at 7 and 11 years of age revealed that the eGFR was lower in these children at 11 years of age and that the difference was statistically significant (*p* = 0.0002). Calculation of eGFR was based on cystatin C levels, which were measured in both age groups.

### Results of renal ultrasound

Mean values of all renal measurements were significantly lower in the ELBW group compared to the term control group at 11 years of age, and this was seen at 7 years of age as well, as shown in Table 6. In 19 children from the study group relative kidney volume was less than 45 ml/m^2^ at 11 years of age. This difference was statistically significant when compared to the control group at the same age. A difference between those two groups was also observed at the age of 7 years. Asymmetric kidneys were found in 8 children from the ELBW group, compared to 3 in the control group at 11 years of age (*p* = 0.7). At the time of the first follow-up we observed asymmetric kidneys in 12 ELBW children and 1 term child (*p* = 0.02). These results are shown in Table 3.

**Table 6:** Comparison of the results of ultrasonographic measurements between extremely low birth weight (ELBW) children and control children

Ultrasonographic measurements	First follow-up (at age 7 years)			Second follow-up (at age 11 years)		
	Control (full term) group (*n* = 36)	Study (ELBW) group (*n* = 64)	*p* ^a^	Control (full term) group (*n* = 36)	Study (ELBW) group (*n* = 64)	*p* ^a^
Absolute right kidney length (cm)	8.07 ± 0.65	7.35 ± 0.65	<0.01	8.97 ± 0.95	8.29 ± 0.89	<0.01
Absolute left kidney length (cm)	8.06 ± 0.72	7.37 ± 0.72	<0.01	9.04 ± 0.99	8.39 ± 0.89	<0.01
Absolute right kidney volume (ml)	58 ± 14	40 ± 10	<0.01	75 ± 20	59 ± 15	<0.01
Absolute left kidney volume (ml)	57 ± 15	41 ± 11	<0.01	78 ± 20	64 ± 17	<0.01
Relative right kidney length (%)	99.8 ± 6	96.6 ± 7	0.02	98.2 ± 8	93.1 ± 9	<0.01
Relative left kidney length (%)	98.4 7	95.6 ± 7	0.08	97.3 ± 9	92.9 ± 8	<0.01
Relative right kidney volume (ml/m^2^)	61.2 ± 11	50.8 ± 10	<0.01	58.2 ± 10	51.4 ± 10	<0.01
Relative left kidney volume (ml/m^2^)	60.3 ± 12	51.4 ± 12	<0.01	60.7 ± 10	55.2 ± 12	0.02

Data are presented as the mean ± SD

^a^ *p* value according to Student’s *t* test

## Discussion

The focus of this study was an analysis of the renal function of children born with ELBW in comparison to that of children born full term, at 11 years of age. We observed and compared the renal function of the same cohort of children for 4 years [31] and assessed the growth of the kidney over that same period of time. The children who participated in our multicentre study come from three tertiary referral centres in the Malopolska region of Poland, which makes the study cohort complete and representative, with a high percentage of observation (up to 82 % of the 7-year-old cohort). We used anthropometric standards based on the Polish population [32]. Renal ultrasound standardization was crucial for the reliability of the study. Based on the observation that ELBW children were shorter and weighed less than children in the control group, we therefore corrected kidney measurements to each child’s height (kidney length) and BSA (kidney volume). Renal ultrasound examinations were performed by the same certified examiners, using the same protocol in both follow–up evaluations. The same outcomes and cut-off points in measurements were set in the assessment of kidney function and growth in both groups of children at 7 and 11 years of age.

One of the greatest strengths of our study is the evaluation of the same cohort over a 4-year period, and we believe that a major limitation is that the cohort of ELBW children was incomplete at the examination at 11 years of age. Those children who missed the second follow-up assessment were the same (as a group) in terms of gestational age, maturation, premature birth complications and renal findings at the age of 6–7 years (data on request).

### Renal size

In our study, renal ultrasound revealed that the ELBW children had significantly smaller renal volumes at 7 and 11 years of age that the age-matched term controls. Kaijzer-Veen et al. [30] evaluated young adults (20 years of age) who had been born very preterm (<32 weeks of gestation), either small or appropriate for gestational age (SGA and AGA, respectively). This 20-year observation period is the longest reported to date. Their analysis revealed that both the absolute and relative kidney length and volume were significantly lower in SGA and AGA children (in particular, the left kidney and in women) than young adults born full term. However, their data consisted of just a single measurement taken at 20 years of age; no other measurements were obtained during the childhood period. In comparison to the study population of Kaijzer-Veen et al. [30], the preterm subjects in our study were more immature (in terms of gestational age, up to 81 % were born at <30 weeks of gestation) and had a lower birth weight {<1500 g (Kaijzer-Veen et al.’s study [30]) vs. <1000 g (our study)}. Different conclusions were drawn Rakow et al. [36] who found that the total renal volume correlated with birth weight but did not observe significant differences with in terms of renal function or volume between preterm, term SGA and term AGA groups. The authors assessed children born preterm at <32 gestational weeks, term but small for gestational age (SGA) and term appropriate for gestational age (AGA) at 9–12 years of age. However, as mentioned above, the children assessed in our study were more immature at birth.

### Renal function

Our study revealed that renal function was impaired in our study group of ELBW children. At 7 years of age, cystatin C levels were within the normal range in all children of this group, but at the second follow-up, cystatin C levels were above the normal range in six 11-year-old ELBW children, although the difference did not reach statistical significance. It should be emphasized that cystatin C levels did change over the 4-year follow-up period, which demonstrates that there is a tendency toward worsening renal function in ELBW children with age and suggests that such children need further observation. All children with increased cystatin C levels were referred to the pediatric nephrology department for further counselling. As an additional parameter we calculated the eGFR in both groups based on cystatin C levels. Creatinine levels in the study which took place at 7 years of age were not measured so we could not calculate eGFR using standard formulas when comparing both age groups. However, there are several studies that have found that both cystatin C and creatinine levels correlate with eGFR in adults and in children [37–39]. Bacchetta et al. showed that cystatin C-derived formulas for estimating eGFR seemed to be accurate in non-selected paediatric patients [40]. Furthermore, serum creatinine level (but not cystatin C level) has been found to correlate with the lean mass of the body. Therefore, cystatin C seems to be a more adequate alternative for the assessment of renal function [41]. Moreover, in ELBW children who were shorter and thinner at follow-up, cystatin C concentrations may reflect eGFR even more accurately than serum creatinine levels [42]. Our study demonstrated that ELBW children had a significantly lower eGFR at 7 and 11 years of age compared to term children of the same age. We found that the eGFR had decreased over the 4-year observation period and that it was significantly lower at 11 years of age than at 7 years of age. However, the study by Rakow et al. found no significant differences in renal function between preterm and term groups (AGA and SGA) [36]. It is important to note that their study focused on a high-risk population with multi-systemic complications that could, at least potentially, affect renal function. Our first follow-up study at 7 years of age showed that SGA and poor weight gain during NICU hospitalization stay were risk factors for renal complications in the ELBW group [31]. Kist-van Holthe et al. [26] evaluated 42 children at the age of 7.5 (±1.0) years who had been born preterm at a gestational age of <32 weeks with nephrocalcinosis. These authors reported that more preterm children with neonatal nephrocalcinosis had mild chronic renal insufficiency (defined as GFR of <85 ml/min/1.73 m^2^) in comparison to term and preterm children without nephrocalcinosis. The study shows that prematurity per se is associated with high blood pressure and relatively small kidneys despite nephrocalcinosis [26].

In conclusion, our data demonstrate that both kidney size and function were affected by preterm birth in children with ELBW and that this trend continued during the 4-year observation period. It would appear that the kidneys of ELBW children are programmed to be smaller and that renal function deteriorates with time. Continued follow-up of these patients will show whether this observed worsening in renal function will persist into adulthood.
